# A PPO Promoter from Betalain-Producing Red Swiss Chard, Directs Petiole- and Root-Preferential Expression of Foreign Gene in Anthocyanins-Producing Plants

**DOI:** 10.3390/ijms161126011

**Published:** 2015-11-12

**Authors:** Zhi-Hai Yu, Ya-Nan Han, Xing-Guo Xiao

**Affiliations:** State Key Laboratory of Plant Physiology and Biochemistry, College of Biological Sciences, China Agricultural University, Beijing 100193, China; 123keyan@163.com (Z.-H.Y.); xxruoer@163.com (Y.-N.H.)

**Keywords:** anthocyanin-producing plant, betalain-producing plant, *Beta vulgaris* subsp. *cicla*, development stage, petiole-preference, polyphenol oxidase (PPO), promoter, root-preferential expression, vegetative organ

## Abstract

A 1670 bp 5′-flanking region of the polyphenol oxidase (PPO) gene was isolated from red Swiss chard, a betalain-producing plant. This region, named promoter *BvcPPOP*, and its 5′-truncated versions were fused with the GUS gene and introduced into Arabidopsis, an anthocyanins-producing plant. GUS histochemical staining and quantitative analysis of transgenic plants at the vegetative and reproductive stages showed that *BvcPPOP* could direct GUS gene expression in vegetative organs with root- and petiole-preference, but not in reproductive organs including inflorescences shoot, inflorescences leaf, flower, pod and seed. This promoter was regulated by developmental stages in its driving strength, but not in expression pattern. It was also regulated by the abiotic stressors tested, positively by salicylic acid (SA) and methyl jasmonate (MeJA) but negatively by abscisic acid (ABA), gibberellin (GA), NaCl and OH^−^. Its four 5′-truncated versions varied in the driving strength, but not obviously in expression pattern, and even the shortest version (−225 to +22) retained the root- and petiole- preference. This promoter is, to our knowledge, the first PPO promoter cloned and functionally elucidated from the betalain-producing plant, and thus provides not only a useful tool for expressing gene(s) of agricultural interest in vegetative organs, but also a clue to clarify the function of metabolism-specific PPO in betalain biosynthesis.

## 1. Introduction

Polyphenol oxidases (PPOs) are type-3 copper enzymes that can catalyze the *o*-hydroxylation of monophenols (monophenolase activity, EC 1.14.18.1) and oxidation of *o*-diphenols to *o*-quinones (diphenolase activity, catechol oxidase activity, EC 1.10.3.1). They are widely distributed in plants, animals and microbes. In plants, PPOs can be classified into two groups based on their physiological roles: Group 1: PPOs engaged in adaptation and defense against biotic and abiotic stresses; Group 2: PPOs involved in some specific secondary metabolisms [1,2]. For Group 1 PPOs, their physiological roles have been linked to PPO genes [3,4,5,6,7,8] and a grand body of PPO genes have their promoters cloned and functionally analyzed, such as the PPO promoters in tomato [9,10], eggplant [11], dandelion [12], pineapple [13], rice [14], artichoke [15] and rubber tree [16] *etc*. However, for Group 2 PPOs, their involvement in specific secondary metabolism have been proposed based mainly on biochemical analysis, and only PPOs involved in the biosynthesis of aurones [17,18], lignans [19] and in tyrosine metabolism [20] have been approved by gene functional analysis.

PPO has long been proposed to be involved in the biosynthesis of betalains by sequential hydroxylation of l-tyrosine to form l-DOPA and oxidation of the DOPA to produce dopa-quinone [21,22,23] and this proposition has been widely adopted [24,25,26,27,28,29]. However, the molecular mechanisms in support of this hypothesis, to our knowledge, remain unclear. Joy and colleagues [30] cloned and sequence-characterized two PPO genes from *Phytolacca Americana*, but did not report their function. Kaneda and co-workers [31] isolated five PPO genes from *Portulaca oleracea*, and showed that two of them, PoPPO2 and PoPPO5 could catalyze hydroxylation of octylphenol, nonylphenol and 17β-estradiol. Whether these two PPOs and the rest can mediate conversion of l-tyrosine remains unknown. Our team purified and characterized a PPO from red Swiss chard (*Beta vulgaris* subspecies *cicla*), a betalain-rich vegetable [32] and cloned a PPO gene [33]. In this study, we report isolation, sequence and functional analysis of this PPO gene’s promoter, together with its 5′-deleted versions. This promoter, named *BvcPPOP*, directs GUS gene expression in the vegetative organs of the transgenic anthocyanin-producing plant, Arabidopsis, with a root- and petiole-preferential pattern. The 5′-deleted versions did as well, but at different levels. The information reported here may help to understand whether the PPO is engaged in betalain biosynthesis, at least in red Swiss chard, and the promoter and its 5′-deleted versions disclosed here may provide useful tools for crop improvement via biotechnology route.

## 2. Results

### 2.1. Cloning and Sequence Analysis of the BvcPPOP Promoter

**Figure 1:**
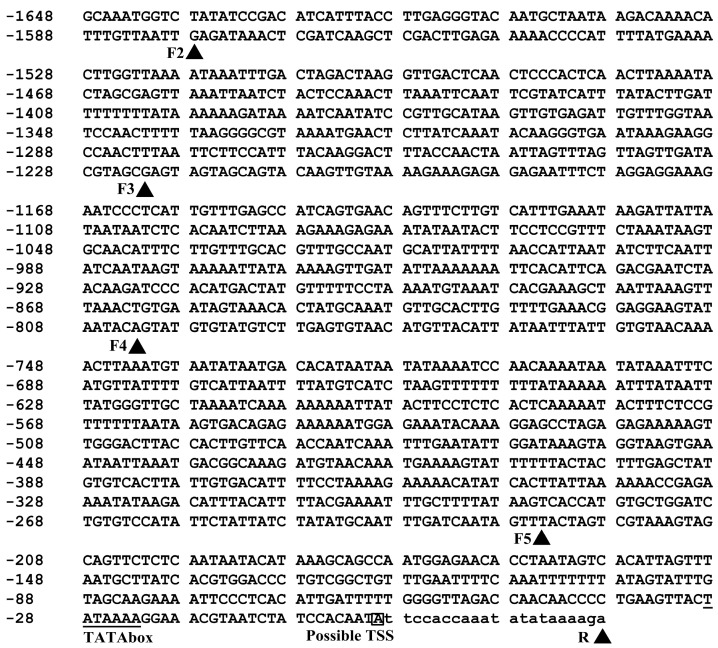
Nucleotide sequence of the 5′-flanking region of *PPO* gene. Nucleotides are numbered on the left with the possible transcription start site which is designated as +1 and white-boxed. The TATA-box is underlined. The deletion positions are indicated with a black triangle behind the short name of forward (F2 to F5) and reverse (R) primers.

A 1670-bp promoter region was successfully amplified using the PCR method and deposited in GenBank (accession No. KT285044). In this region, a transcription start site (TSS), a TATA-box which is 22 bp upstream of the TSS (Figure 1) and a large number of other cis-acting elements (Table S1) were predicted based on New PLACE [34] and PlantCARE [35] analysis.

### 2.2. BvcPPOP Directed Petiole- and Root-Preferential Expression of GUS Gene at the Vegetative Stage of Transgenic Arabidopsis

**Figure 2:**
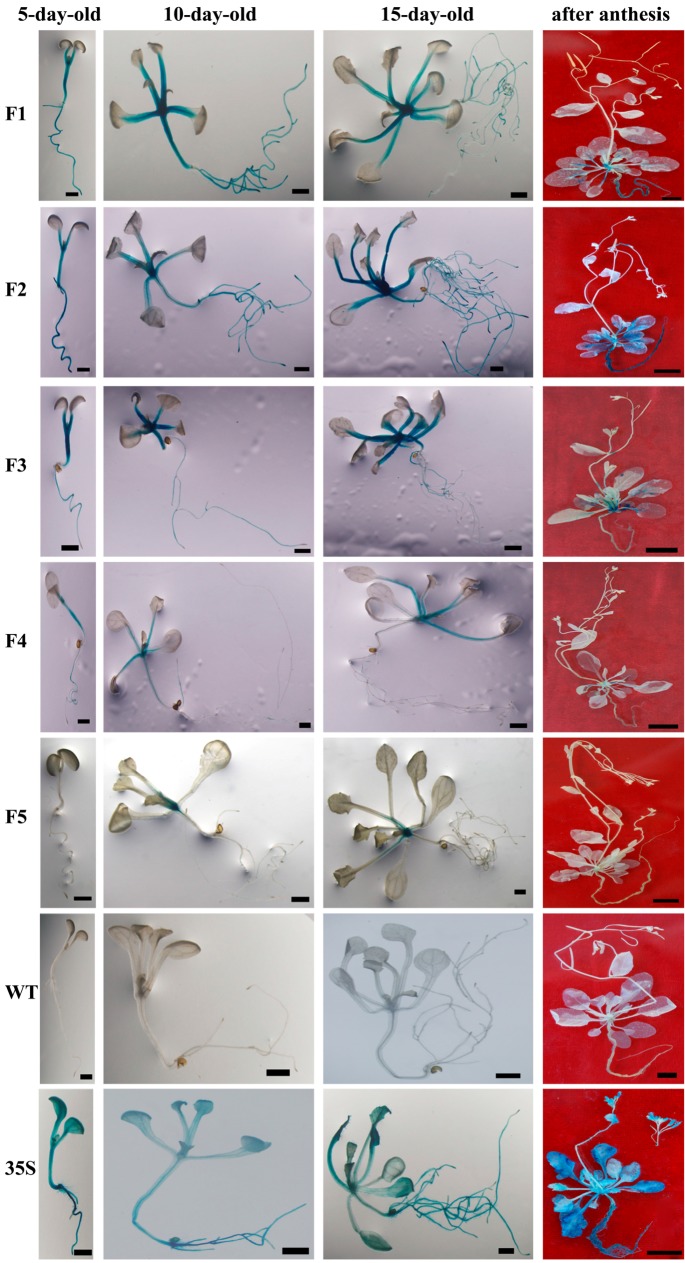
GUS staining of T_3_ Arabidopsis. Bar = 1 mm, Bar = 1 cm (“after anthesis”). For each construct, three independent lines (more than 20 plantlets of each line) were GUS-stained; representative plants are shown.

**Figure 3:**
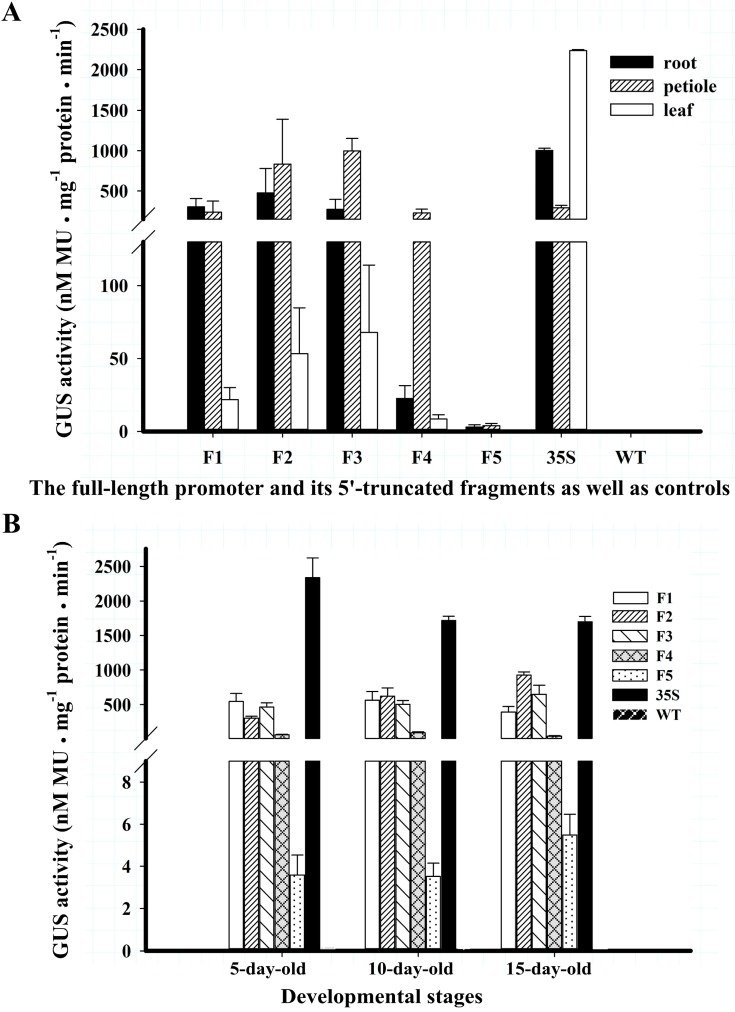
The GUS activities of T_3_ Arabidopsis. Data are shown as mean values of three independent lines of each construct ± SD. (**A**) The GUS activity measured in root, petiole and leaf of 15-day-old different construct transgenic seedlings; (**B**) GUS activity driven by serial 5′-truncated *BvcPPOPs* and *CaMV 35S* as well as WT control in different developmental stages. GUS activity of single line of each construct was present in Figure S3.

As shown in F1 of Figure 2, the GUS gene driven by the full-length *BvcPPOP* promoter (F1) was expressed very strongly in the root, hypocotyl, petiole and leaf base of transgenic plantlets aged 5 to 10 days. As plantlets grew (15-day-old), the GUS blue became lighter in some fibrous roots (F1 in Figure 2) except the root-tip (Figure S1), whereas the GUS staining in the petiole, hypocotyl and leaf base remained visibly unchanged, with much stronger staining in root and petiole than in leaf blade. Measured at 15-day-old, the GUS enzymatic activities in the root and petiole were 13-fold and 10-fold higher than that in the leaf, respectively (F1 in Figure 3A). In contrast, the GUS activity of the leaf in *35S-GUS* transgenic plants was higher than that of root and petiole (35S in Figure 3A). At the whole plant level, the GUS activity in *F1-GUS* plants was significantly lower than that in *35S-GUS* plants, being approximately 30% (5-day-old), 40% (10-day-old), and 25% (15-day-old) of that in *35S-GUS* plants (Figure 3B). However, this difference was negligible in petioles (Figure 3A).

### 2.3. BvcPPOP-Directed GUS Expression Is Regulated by Developmental Stages

The expression pattern of the GUS gene driven by *BvcPPOP* changed when the plants entered the reproductive stage. At flowering and after anthesis, the GUS blue in the hypocotyl disappeared completely and that in petiole and the main veins of the rosette leaf faded progressively, although the GUS-staining in root remained strong (“after anthesis” in Figure 2, and GUS activity in Figure S2). In the reproductive organs of broad-sense, such as inflorescence shoot, inflorescence leaf, flower, pod and seed, no GUS blue was visible.

### 2.4. BvcPPOP-Directed GUS Expression Is Differently Regulated by Abiotic Stressors

**Figure 4:**
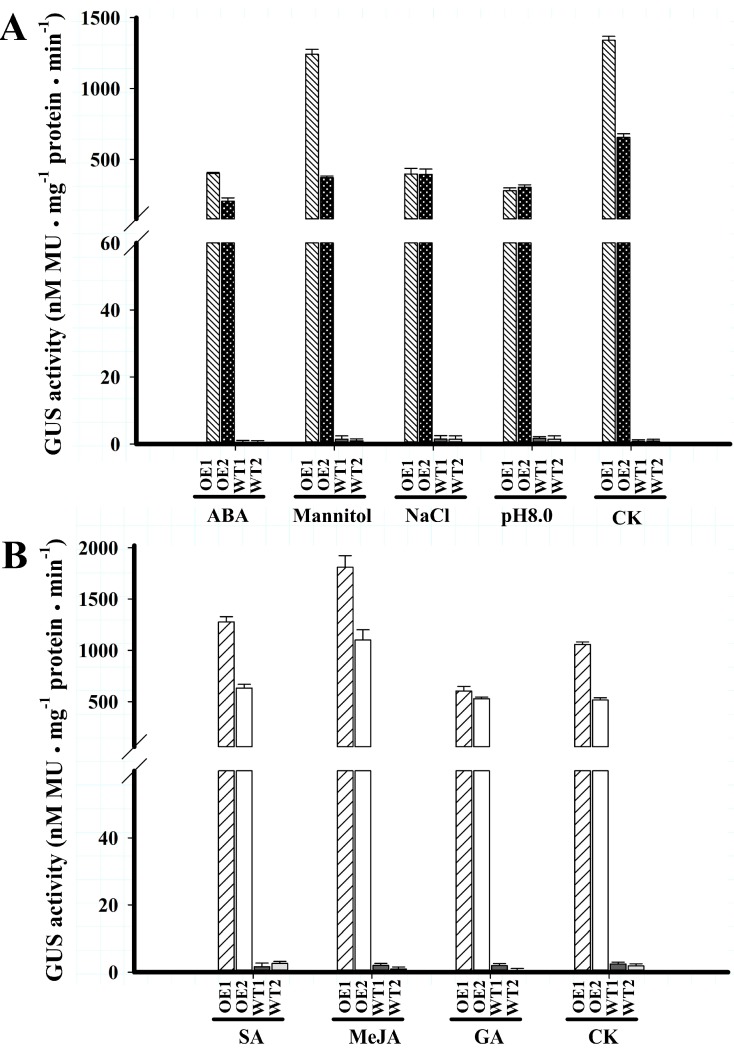
GUS activities of T_3_ seedlings of Arabidopsis plant transformed with full-length *BvcPPOP* in the presence of abiotic stress signals. Data are shown as the mean value ± SD of three replicates of each *BvcPPOP-GUS* transgenic line. (**A**) Set 1 abiotic stress signals; (**B**) Set 2 abiotic stress signals.

*BvcPPOP*-directed *GUS* expression was down-regulated by abiotic stressors tested in Set 1. NaCl (120 mM) treatment of 3-day-old *F1-GUS* transgenic seedlings for one week, resulted in GUS activity decline by around 79%, compared with that untreated, and the ABA (2 µM) or OH^−^ (pH 8.0) decreased about 70%, 60% of the GUS activity, respectively, while mannitol (200 mM) resulted in only an approximately 19% decrement of the activity (Figure 4A).

The regulation of *BvcPPOP*-directed GUS expression was varied in Set 2 stressors. SA (1 mM) and MeJA (100 µM) root-treatment of 10-day-old plantlets for 24 h increased GUS activity by 21% and 84%, respectively, whereas GA (100 µM) decreased the activity by nearly 28% (Figure 4B), compared with water treatment (CK).

### 2.5. 5′-Truncated BvcPPOPs Display Various Expression Patterns and/or Levels in Transgenic Arabidopsis

5′-Deletion of 70 bp at the far end of F1 (*BvcPPOP*) did not change the expression pattern of the GUS gene under its control at vegetative stages of transgenic Arabidopsis, but enhanced GUS staining and enzymatic activity in the root and petiole, in particular for 15-day-old seedlings and in petiole (F1 and F2 in Figure 2 and Figure 3). This enhancement resulted in a higher GUS activity in the petiole than that in root (Figure 3A). Although this enhancement extended to reproductive stages in the root and main veins of rosettes leaves, no stronger GUS-blue was noted in petiole (F1 and F2 in Figure 2). At reproductive stages, the *F2-GUS* plant showed light GUS blue in inflorescence shoot, but no GUS blue was visible in inflorescence leaf, flower, pod and seed.

Further 5′-deletion of 356 bp weakened GUS blue and enzymatic activity in the root of 5 to 10-day-old seedlings (F2 and F3 in Figure 2 and Figure 3), and no GUS blue was visible even at the root-tip of 15-day-old plants (Figure S1). However, GUS blue and enzymatic activity were more or less enhanced in the petiole of *F3* (−1222 to +22)-*GUS* plants. At reproductive stages, the GUS staining in the inflorescence shoot remained unchanged, whereas that in the root and main veins of rosette leaves was significantly weakened (F2 and F3 in Figure 2).

Dramatic decline in GUS blue and enzymatic activity was noted in *F4* (−803 to +22)-*GUS* plants from the vegetative stage to reproductive stage, but no change in *GUS* expression pattern was observed (F3 and F4 in Figure 2 and Figure 3), in comparison with the *F3-GUS* plant.

The weakest GUS blue was displayed in *F5* (−225 to +22)-*GUS* seedlings. Although no GUS-blue was visible in 5-day-old plantlet, a faint GUS-blue was observed in the petiole and root of 10-day and older plants (F5 in Figure 2), and the GUS activity was detected (F5 in Figure 3). It is worth noting that at this vegetative stage, and even at such a low GUS activity, the *GUS* expression pattern remained unchanged, compared with F4. At reproductive stages, the GUS-blue weakened more and was visible only in the base of petiole and some sections of root (F5 in Figure 2).

## 3. Discussion

A 1670 bp 5′-flanking region of a PPO gene was cloned from red Swiss chard, a betalain-producing plant (Figure 1), and as predicted, this region could direct expression of the foreign gene (*GUS*) in transgenic Arabidopsis, a non-betalain-producing plant (F1 in Figure 2), indicating its role as promoter. This promoter was named *BvcPPOP*.

Different from *CaMV 35S* promoter, the *BvcPPOP* (F1) drove expression of *GUS* in vegetative organs, but not or almost not in broad sense reproductive organs such as inflorescence shoot, inflorescence leaf, flower, pod and seed (F1 *vs.* 35S in Figure 2). Even among the vegetative organs, *BvcPPOP*-driven *GUS* expression was not equal. It expressed preferentially in root and petiole at the vegetative stage (F1 in Figure 2 and Figure 3). Furthermore, its expression was regulated developmentally. As plantlets growing and entering reproductive stage, the GUS activity in hypocotyl, petiole and main veins of rosette was almost absent or decreased significantly, although it remained unchanged in root (F1 in Figure 2).

In non-betalain plants, the *PPO* promoters were reported to be inducible by abiotic stress signals. For example, *PINPPO1* promoter was induced by GA [13] and those of eggplant by MeJA and/or SA [11]. Our *BvcPPOP* cloned from the betalain plant, red Swiss chard, was also responsive to various abiotic signals tested. It was down-regulated by GA, ABA, NaCl and OH^−^ (pH 8.0) but up-regulated by SA and MeJA (Figure 4). This suggested that the native gene *PPO* under its control might be inducible by some abiotic stresses. What is worth to note is that these regulations were restricted to the expression strength, but not pattern.

In order to obtain the minimal promoter and elucidate the cis-acting elements controlling root- and/or petiole-preferential expression, we performed a set of 5′-deletions of *BvcPPOP*. The results showed that deletion of 70 bp at the 5′-extreme (from −1648 to −1579, noted as “F2”) did not affect GUS expression pattern, but the expression strength in the petiole was increased and became higher than in root (F2 *vs.* F1 in Figure 2 and Figure 3B). This means that in the 70 bp fragment there may exist some kind of weak petiole-specific suppressor, which remains to be identified. Further deletion of 356 bp (from −1578 to −1223, noted as “F3”) decreased more or less *GUS* expression strength in root, and in particular at the root tip and at reproductive stages, but not the expression pattern (F3 *vs.* F2 in Figure 2 and Figure 3B, Figure S1). In this region, there may be weak root-specific suppressor(s) that are up-regulated by the developmental stage. Apart from the suppressor(s), all cis-elements (Table S1) in this region seem functionally redundant. In contrast, deletion of the next 419 bp region (between −1222 bp and −804 bp) resulted in a dramatic decrement in *GUS* expression strength, although the expression pattern remained unchanged (F4 *vs.* F3 in Figure 2 and Figure 3). Accordingly, in this 419 bp region, there might exist some kind of “maintainer” or “enhancer” for gene expression. If the “maintainer” or “enhancer” was present, its function was much more pronounced in the region of −803 to −226, because deletion of this region left a very weak *GUS* expression and only in the petiole and root of 10-day and older plants at vegetative stages, and in the base of petiole and some sections of root at reproductive stages (F5 *vs.* F4 in Figure 2 and Figure 3). Interestingly, after a set of 5′-deletions, the rest of the region (−225 to + 22) of the *BvcPPOP* retained the capacity not only to drive *GUS* expression, but also to direct root- and petiole-preferential expression. Whether the root- and petiole-preferential expression is decided by cis-acting element(s) located upstream of the TATA-box or by 5′-UTR in this region remains to be elucidated, and which is under the progress.

**Figure 5:**
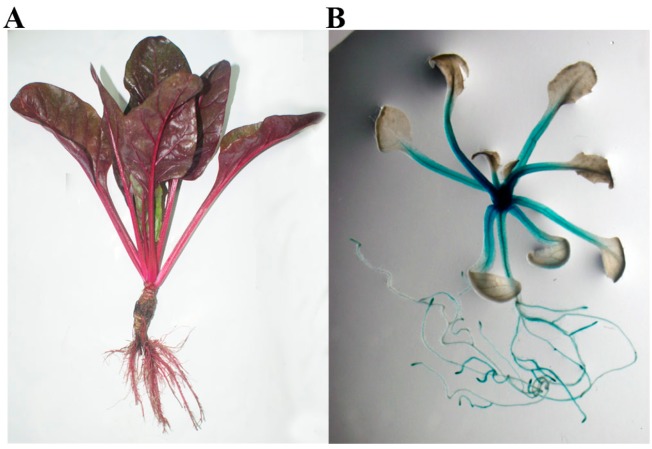
Comparison of betalain distribution and/or accumulation in young red Swiss chard with GUS staining pattern in young plants of *BvcPPOP-GUS* transgenic Arabidopsis. (**A**) Young red Swiss chard; (**B**) young plant of *BvcPPOP-GUS* transgenic Arabidopsis.

It is well known that betalains seem inert to move from the tissues and/or organs where they are synthesized [32,36], and their synthesis is assumed to start by PPO-type tyrosinase-mediated tyrosine hydroxylation [24,26,28,29,37]. Thus, where the betalains accumulated and/or distributed must be the place where the promoter of gene(s) responsible for key enzyme(s) are involved in betalain biosynthesis and performs its function. In young red Swiss chard, the betalains are accumulated and/or distributed mainly in the root and petiole (Figure 5), and similarly, the strong GUS-blue appears essentially in the root and petiole in young plants of *BvcPPOP-GUS* transgenic Arabidopsis (Figure 5). This similarity or agreement implies that our cloned promoter *BvcPPOP* as well as its native gene *BvcPPO* might be involved in betalain biosynthesis.

PPO’s involvement in plant betalain biosynthesis is a long-standing hypothesis [24,26,28,29,37] and this hypothesis was challenged by the discovery of a new P450 (CYP76AD1) which could catalyze conversion of l-DOPA to cyclo-DOPA in sugar beet [36]. Hatlestad and co-workers (2012) even ruled out the involvement of conventional PPO and laccase based on relatively low abundance of the transcripts in sugar beet [36]. If this inference is correct in sugar beet, and applicable to red Swiss chard, our cloned promoter *BvcPPOP* may not be the promoter of such a conventional *PPO*, but that of secondary metabolism-specific *PPO* (Group 2 PPO) as described in Mayer, 2006 [38]; Thipyapong *et al.* 2007 [1] and Sullivan 2014 [2]. Thus, possible involvement of metabolism-specific PPO in betalain biosynthesis, especially in the first step and in the betaxanthin biosynthesis would be worth investigating.

In summary, a full-length *PPO* promoter (*BvcPPOP*) was cloned, for the first time, from a betalain-producing plant and this promoter could direct the GUS gene to express in the vegetative organs of an anthocyanin-producing plant, Arabidopsis, with a root- and petiole-preferential pattern. The promoter was regulated by developmental stage in its driving strength, but not its expression pattern. It was also regulated by various abiotic stressors tested. Its 5′-truncation versions varied remarkably in the driving strength, but not obviously in expression pattern, and even a short region from −225 to +22 retained root- and petiole-preferential expression pattern. This expression pattern is highly similar with that of betalain accumulation and/or distribution of red Swiss chard from which the promoter was cloned, implying possible involvement of the promoter’s native gene in betalain biosynthesis, and particularly in the first step of the pathways and in betaxanthin biosynthesis. Successful cloning and functional elucidation of this *PPO* promoter from betalain-producing plants provides not only a useful tool for expressing gene(s) of agricultural interest in vegetative organs, but also an important clue to clarify the function of metabolism-specific PPO in betalain biosynthesis, which is still unclear.

## 4. Experimental Section

### 4.1. Plant Materials and Growth Conditions

Seeds of red and green Swiss chard (*Beta vulgaris* subspecies *cicla*) were kindly provided by Prof. Xi-Xiang Li of the Institute of Vegetables and Flowers, CAAS, China. Seeds were germinated in a plant growth incubator and then the seedlings were transplanted to pots in a greenhouse with natural light and temperature fluctuated from 20–25 °C during the day to 15–20 °C at night. Seeds of *Arabidopsis thaliana* (Columbia-0 ecotype) were surface-sterilized with 0.5% NaClO for 10 min and then rinsed five times with sterile water. The sterilized seeds were placed on MS medium [39]. Plates were transferred to a plant growth incubator for seed germination after vernalization at 4 °C for 3 days. The seedlings were planted in a culture room at 22 ± 1 °C with a photoperiod of 16-h light/8-h dark.

### 4.2. Isolation of the Promoter and Its Deletion Analysis

Genomic DNA was extracted from fresh leaves of red Swiss chard according to an improved CTAB (cetyltrimethylammonium bromide) method [40]. The promoter region was cloned by PCR with primer pair FW1/RW (Table 1), and the PCR products were inserted into pGEM-T vector (Promega, Madison, WI, USA) and sequenced. The resulted fragment, named *BvcPPOP*, was designed as full-length promoter (F1).

A set of 5′-deletions of F1 were generated by PCR using primer pairs FW2~FW5/RW (Table 1), and the truncated promoter fragments were also linked into the pGEM-T vector and sequenced as described above.

**Table 1:** Oligonucleotide primers used for PCR cloning and deletion of *BvcPPOP* promoter.

Primer Name	Primer Sequence (5′ to 3′) *
*Forward*	
FW1	AAGCTTGCAAATGGTCTATATCCGAC
FW2	AAGCTTGAGATAAACTCGATCAAGCTCG
FW3	AAGCTTGAGTAGTAGCAGTACAAGTTG
FW4	AAGCTTAGTATGTGTATGTCTTGAGTG
FW5	AAGCTTTACTAGTCGTAAAGTAGCAGTTCTC
*Reverse*	
RW	GGATCCTCTTTTATATATTTGGTGGA

* Underlined are *Hind* III and *Bam* HI sites at the forward and reverse primers, respectively.

### 4.3. Plant Expression Vector Construction and Agrobacterium-Mediated Transformation

The sequencing-verified promoter fragments were took out from their corresponding pGEM-T vector with *Hind* III and *Bam* HI and then individually cloned into a binary vector pBI121 (Clontech, Mountain View, CA, USA) to replace CaMV 35S promoter in front of β-glucuronidase (GUS) gene, which gave rise to pBvcPPOPn-GUS vectors (here *n* = 1 to 5).

All constructs including *CaMV35S-GUS* (original pBI121) were transferred into the *Agrobacterium tumefaciens* strain GV3101 by the freeze-thaw procedure [41]. Arabidopsis plants were transformed by using the floral dip method [42] and the T_1_ transformants were selected on MS medium containing 50 mg/L of kanamycin (Kan). Kan-resistant seedlings were PCR verified and those with a strong and sharp PCR band were transplanted in pots and grown in a culture room. The T_3_ generation of three independent lines for each construct was used for subsequent analysis, with wild type as negative controls.

### 4.4. Analysis of GUS Expression

Histochemical GUS staining and quantitative analysis for GUS activity in the transgenic plants were performed according to the method described by Jefferson *et al.* [43] and slightly modified by Han *et al.* [44]. Briefly, the Arabidopsis whole-plantlets of different developmental stages (5-day-old, 10-day-old, 15-day-old, after anthesis) were incubated at 37 °C for 12 to 16 h in GUS staining solution (50 mM phosphate buffer, pH 6.7, 1 mM EDTA pH 8.0, 0.2% (*v*/*v*) Triton-100, 1 mM K_3_FeCN_6_, 1 mM K_4_FeCN_6_, 0.5 mg/mL 5-bromo-4-chloro-3-indoxyl-d-glucuronic acid (X-gluc)). After staining, the samples were cleared in GUS destaining solution (75% ethanol, 25% acetic acid) for more than 1 h at room temperature, and then photographed using an Olympus SZX16-DP72 stereomicroscope. For stained plants of after-anthesis, they were photographed using a digital camera (Sony, DSLR-A350, Tokyo, Japan).

For quantitative GUS activity assay, the samples were prepared as previously described [45], and the enzymatic reaction was carried out according to Han *et al.* [44].

### 4.5. Abiotic Stress Treatment

To investigate the response of *BvcPPOP* to different abiotic stress signals, two independent *F1-GUS* transgenic lines (T_3_ generation) were subjected to abiotic stressors. The stressors were divided in two sets: 1: NaCl, abscisic acid (ABA), mannitol, and OH^−^; 2: gibberellin (GA), methyl jasmonate (MeJA) and salicylic acid (SA). For Set 1 stressor treatment, the disinfected seeds were vernalized at 4 °C for 3 days and then placed in a culture room with a photoperiod of 16-h light/8-h dark. Three-day-old plantlets of each line with a uniform growth were transplanted on the normal MS medium supplying with or without 120 mM NaCl, 2 µM ABA or, 200 mM mannitol or transplanted on higher-pH MS medium (pH 8.0). Seven days later, plants were sampled for protein extraction. For Set 2 stressor treatment, the roots of 10-day-old seedlings were immersed in water solution supplemented with or without 1 mM SA, 100 µM MeJA or 100 µM GA for 24 h [46]. GUS activity assay was performed as described above. The wild type plantlets were treated in parallel as negative controls. The mean values of two lines were used to estimate their regulation strength.

## 5. Conclusions

Red Swiss chard *PPO* promoter, *BvcPPOP*, with its 5’-truncted versions could direct expression of foreign gene(s) in vegetative organs with root- and petiole-preference, and this expression pattern is consistent with betalains accumulation/distribution in red Swiss chard. This promoter with its derivatives may be used in the gene-engineering for crop improvement and in the study of betalain biosynthesis.

## Supplementary Materials

Supplementary materials can be found at http://www.mdpi.com/1422-0067/16/11/26011/s1.
