# Supplementary material for: A PPO Promoter from Betalain-Producing Red Swiss Chard, Directs Petiole- and Root-Preferential Expression of Foreign Gene in Anthocyanins-Producing Plants
Source: Int J Mol Sci. 2015 Nov 12;16(11):27032–43. doi: 10.3390/ijms161126011 (PMC4661869; doi:10.3390/ijms161126011)
Supplement: Supplementary file 1 [file ijms-16-26011-s001.pdf]

Supplementary Materials: A PPO Promoter from Betalain-Producing Red Swiss Chard, Directs Petiole- and Root-Preferential Expression of Foreign Gene in Anthocyanins-Producing Plants

Zhi-Hai Yu, Ya-Nan Han and Xing-Guo Xiao

Table S1. Motif analysis of *BvcPPOP* promoter.

Motif Identity	Motif Sequence	Sense Strand	Antisense Strand
CACTFTPPCA1	YACT	17	16
DOFCOREZM	AAAG	20	6
CAATBOX1	CAAT	16	8
EBOXBNNAPA	CANNTG	10	10
MYCCONSENSUSAT	CANNTG	10	10
GT1CONSENSUS	GRWAAW	11	9
GTGANTG10	GTGA	11	6
ARR1AT	NGATT	4	12
GATABOX	GATA	5	10
POLLEN1LELAT52	AGAAA	10	5
ROOTMOTIFTAPOX1	ATATT	10	4
WRKY71OS	TGAC	7	6
IBOX	GATAAG	7	3
TAAAGSTKST1	TAAAG	6	2
ACGTATERD1	ACGT	6	2
POLASIG3	AATAAT	5	2
IBOXCORE	GATAA	3	4
TATABOX5	TTATTT	2	5
BIHD1OS	TGTCA	4	3
CARGCW8GAT	CWWWWWWWWG	3	3
WBOXHVISO1	TGACT	3	2
WBOXNTERF3	TGACY	3	2
MYBCORE	CNGTTR	1	4
NTBBF1ARROLB	ACTTTA	2	3
RAV1AAT	CAACA	4	1
GT1GMSCAM4	GAAAAA	4	1
CURECORECR	GTAC	2	2
MYBST1	GGATA	1	3
MYBCOREATCYCB1	AACGG	1	3
CCAATBOX1	CCAAT	3	1
GAREAT	TAACAAR	3	1
POLASIG2	AATTAAA	2	1
MYBGAHV	TAACAAA	2	1
AMYBOX1	TAACARA	1	2
TATABOX2	TATAAAT	1	2
OSE2ROOTNODULE	CTCTT	1	2
NODCON2GM	CTCTT	1	2
EECCRCAH1	GANTTNC	2	1
RHERPATEXPA7	KCACGW	3	0
ABRELATERD1	ACGTG	1	2
SEF4MOTIFGM7S	RTTTTTR	1	2
REALPHALGLHCB21	AACCAA	1	1
REBETALGLHCB21	CGGATA	0	2

Table S1. *Cont.*

Motif Identity	Motif Sequence	Sense Strand	Antisense Strand
WBOXATNPR1	TTGAC	2	0
MYB1AT	WAACCA	1	1
REBETALGLHCB21	CGGATA	0	2
LTRECOREATCOR15	CCGAC	1	1
CPBCSPOR	TATTAG	0	2
GT1CORE	GGTTAA	1	1
OSE1ROOTNODULE	AAAGAT	2	0
RBCSCONSENSUS	AATCCAA	2	0
INRNTPSADB	YTCANTYY	1	1
ACGTABOX	TACGTA	1	1
−300ELEMENT	TGHAAARK	2	0
ERELEE4	AWTTCAAA	0	2
ABRERATCAL	MACGYGB	1	1
MYCATERD1	CATGTG	1	1
MYCATRD22	CACATG	1	1
MARTBOX	TTWTWTTWTT	0	2
SV40COREENHAN	GTGGWWHG	0	2
TATCCAOSAMY	TATCCA	1	1
CACGTGMOTIF	CACGTG	1	1
TATABOX3	TATTAAT	0	1
ASF1MOTIFCAMV	TGACG	1	0
CEREGLUBOX2PSLEGA	TGAAAACT	1	0
WBBOXPCWRKY1	TTTGACY	1	0
GCN4OSGLUB1	TGAGTCA	0	1
CAREOSREP1	CAACTC	1	0
BOXCPSAS1	CTCCCAC	1	0
MYB2CONSENSUSAT	YAACKG	0	1
CCA1ATLHCB1	AAMAATCT	0	1
IBOXCORENT	GATAAGR	0	1
MYB1LEPR	GTTAGTT	1	0
CTRMCAMV35S	TCTCTCTCT	0	1
T/GBOXATPIN2	AACGTG	0	1
WUSATAg	TTAATGG	0	1
SP8BFIBSP8BIB	TACTATT	0	1
NAPINMOTIFBN	TACACAT	0	1
L1BOXATPDF1	TAAATGYA	1	0
SEF3MOTIFGM	AACCCA	0	1
SORLIP5AT	GAGTGAG	0	1
SEBFCONSSTPR10A	YTGTCWC	0	1
SREATMSD	TTATCC	0	1
TBOXATGAPB	ACTTTG	1	0
TGTCACACMCUCUMISIN	TGTCACA	0	1
DRE1COREZMRAB17	ACCGAGA	1	0
−10PEHVPSBD	TATTCT	1	0
BOXIINTPATPB	ATAGAA	0	1
CIACADIANLELHC	CAANNNNATC	1	0
ANAERO2CONSENSUS	AGCAGC	1	0

Table S1. *Cont.*

Motif Identity	Motif Sequence	Sense Strand	Antisense Strand
PRECONSCRHSP70A	SCGAYNRNNNNNNN NNNNNNNNNNHD	0	1
IRO2OS	CACGTGG	1	0
DRECRTCOREAT	RCCGAC	0	1
CBFHV	RYCGAC	0	1
PYRIMIDINEBOXOSRAMY1A	CCTTTT	0	1
TATCCAYMOTIFOSRAMY3D	TATCCAY	1	0
TATCCACHVAL21	TATCCAC	1	0
TATAPVTRNALEU	TTTATATA	0	1
TATABOX4	TATATAA	1	0

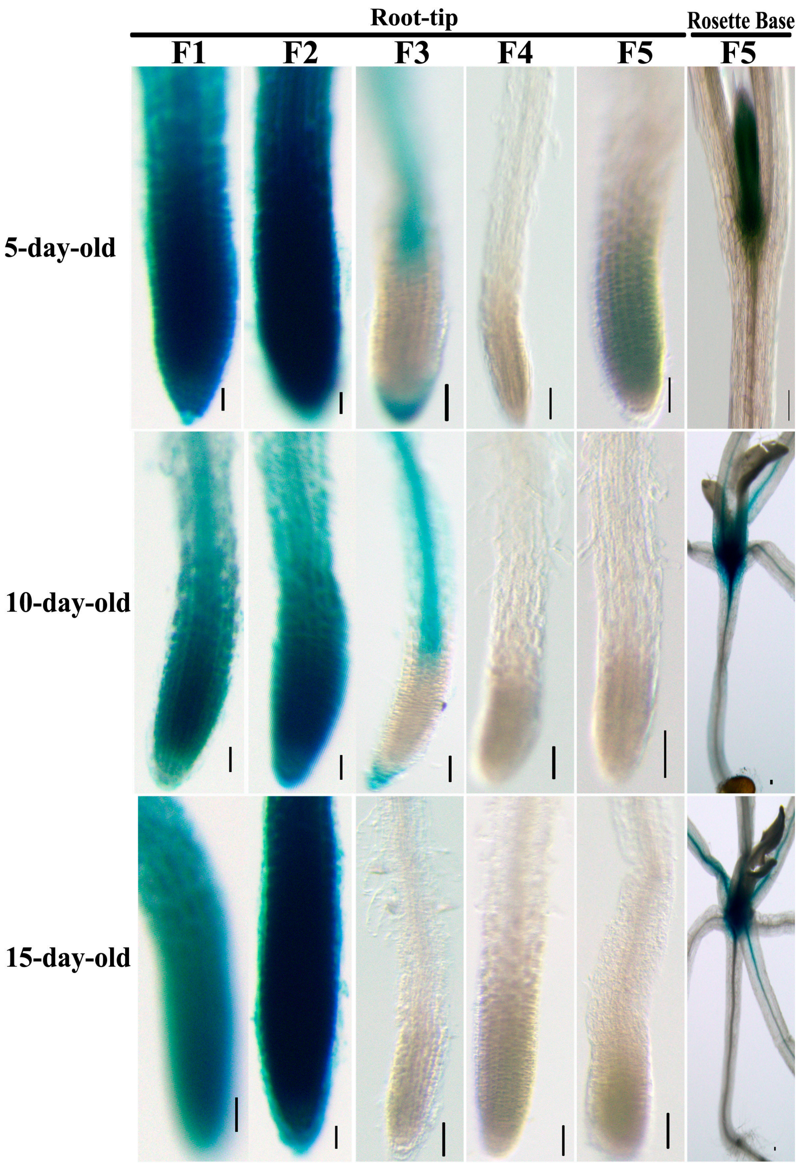

Figure S1. The enlargement of root-tip and rosette base (only for F5) of GUS staining T_3 Arabidopsis. Bar = 100 μm.

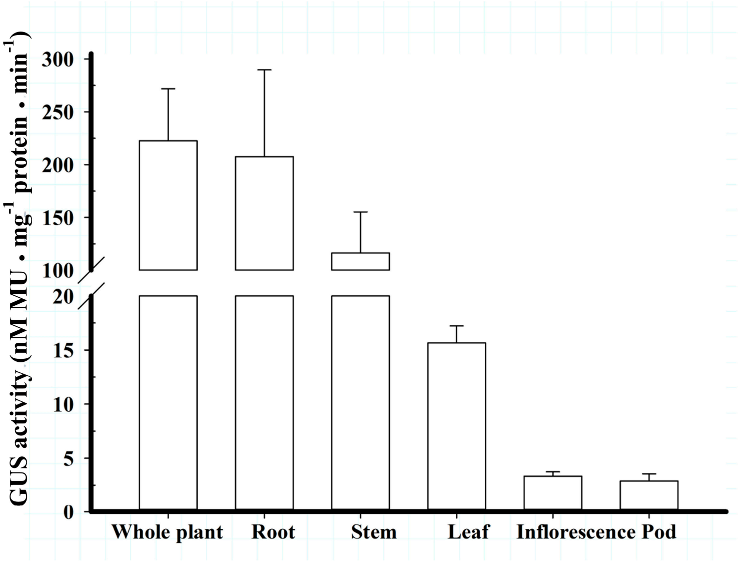

Figure S2. GUS activities of different parts of T_3 *BvcPPOP-GUS* transgenic Arabidopsis after anthesis. Data are shown as the means ± SD (*n* = 3).

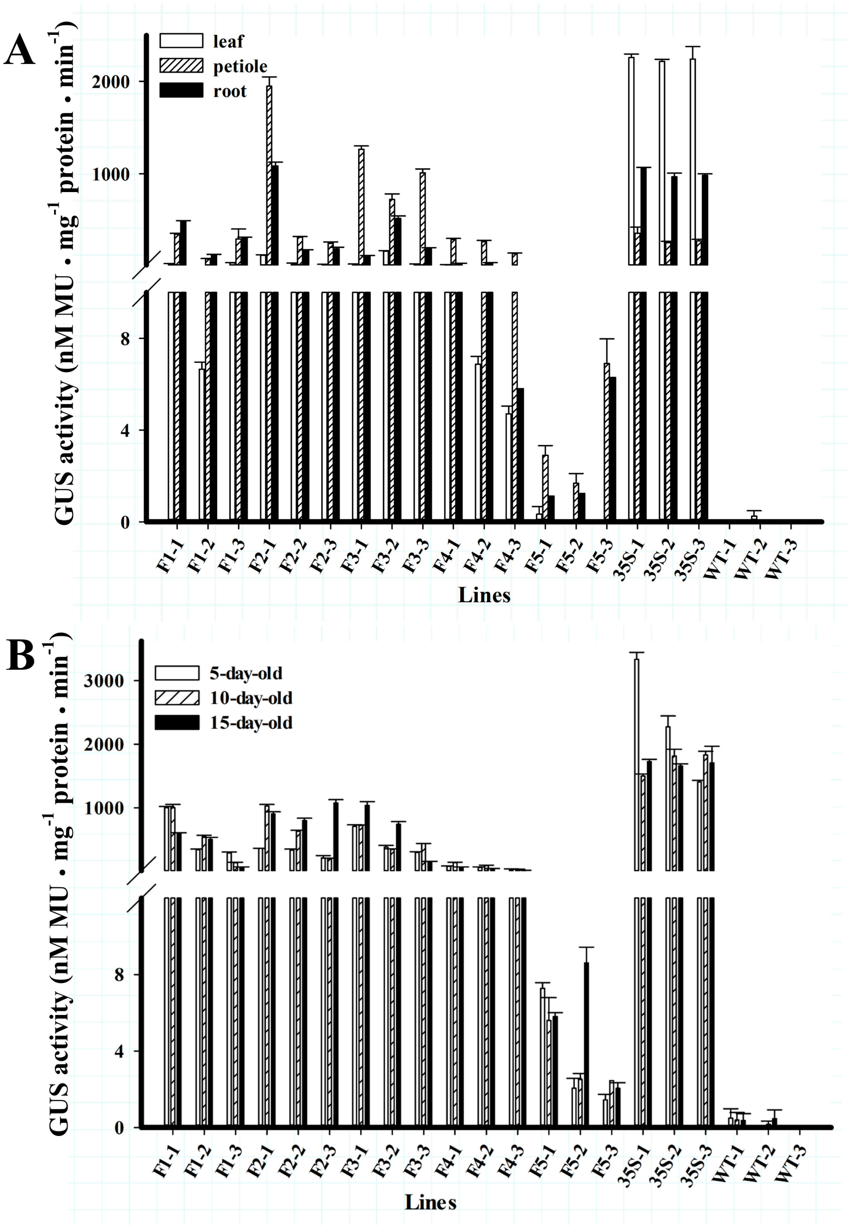

Figure S3. GUS activities of T3 Arabidopsis. Data are shown as mean value of three replicates for each line ± SD. Three independent lines of each construct are present. (**A**) The GUS activity measured in root, petiole and leaf of 15-day-old different constructs transgenic seedlings; (**B**) The GUS activity driven by serial 5′-truncated *BvcPPOP*s and *CaMV 35S* as well as WT control in different developmental stages.
